# Retargeting of microcell fusion towards recipient cell-oriented transfer of human artificial chromosome

**DOI:** 10.1186/s12896-015-0142-z

**Published:** 2015-06-19

**Authors:** Masaharu Hiratsuka, Kana Ueda, Narumi Uno, Katsuhiro Uno, Sayaka Fukuhara, Hajime Kurosaki, Shoko Takehara, Mitsuhiko Osaki, Yasuhiro Kazuki, Yoshikazu Kurosawa, Takafumi Nakamura, Motonobu Katoh, Mitsuo Oshimura

**Affiliations:** Division of Molecular and Cell Genetics, Department of Molecular and Cellular Biology, School of Life Sciences, Faculty of Medicine, Tottori University, 86 Nishi-cho, Yonago, Tottori 683-8503 Japan; Division of Molecular Genetics and Biofunction, Department of Biomedical Science, Institute of Regenerative Medicine and Biofunction, Graduate School of Medical Science, Tottori University, 86 Nishi-cho, Yonago, Tottori 683-8503 Japan; Chromosome Engineering Research Center, Tottori University, 86 Nishi-cho, Yonago, Tottori 683-8503 Japan; Division of Integrative Bioscience, Department of Biomedical Science, Institute of Regenerative Medicine and Biofunction, Graduate School of Medical Science, Tottori University, Yonago, Tottori Japan; Division of Pathological Biochemistry, Department of Biomedical Sciences, School of Life Sciences, Faculty of Medicine, Tottori University, 86 Nishi-cho, Yonago, Tottori 683-8503 Japan; Division of Antibody Project, Institute for Comprehensive Medical Science, Fujita Health University, Toyoake, Aichi 470-1192 Japan; Division of Human Genome Science, Department of Molecular and Cellular Biology, School of Life Sciences, Faculty of Medicine, Tottori University, 86 Nishi-cho, Yonago, Tottori 683-8503 Japan; Japan Science and Technology Agency, CREST, 5, Sanbancho, Chiyoda-ku, Tokyo, 102-0075 Japan

**Keywords:** Human artificial chromosome, Measles Virus fusogenic protein, Chimeric protein

## Abstract

**Background:**

Human artificial chromosome (HAC) vectors have some unique characteristics as compared with conventional vectors, carrying large transgenes without size limitation, showing persistent expression of transgenes, and existing independently from host genome in cells. With these features, HACs are expected to be promising vectors for modifications of a variety of cell types. However, the method of introduction of HACs into target cells is confined to microcell-mediated chromosome transfer (MMCT), which is less efficient than other methods of vector introduction. Application of Measles Virus (MV) fusogenic proteins to MMCT instead of polyethylene glycol (PEG) has partly solved this drawback, whereas the tropism of MV fusogenic proteins is restricted to human CD46- or SLAM-positive cells.

**Results:**

Here, we show that retargeting of microcell fusion by adding anti-Transferrin receptor (TfR) single chain antibodies (scFvs) to the extracellular C-terminus of the MV-H protein improves the efficiency of MV-MMCT to human fibroblasts which originally barely express both native MV receptors, and are therefore resistant to MV-MMCT. Efficacy of chimeric fusogenic proteins was evaluated by the evidence that the HAC, tagged with a drug-resistant gene and an EGFP gene, was transferred from CHO donor cells into human fibroblasts. Furthermore, it was demonstrated that no perturbation of either the HAC status or the functions of transgenes was observed on account of retargeted MV-MMCT when another HAC carrying four reprogramming factors (iHAC) was transferred into human fibroblasts.

**Conclusions:**

Retargeted MV-MMCT using chimeric H protein with scFvs succeeded in extending the cell spectrum for gene transfer via HAC vectors. Therefore, this technology could facilitate the systematic cell engineering by HACs.

**Electronic supplementary material:**

The online version of this article (doi:10.1186/s12896-015-0142-z) contains supplementary material, which is available to authorized users.

## Background

Microcell-mediated chromosome transfer (MMCT) is a technique by which single or small numbers of chromosomes can be transferred from one mammalian cell to another by microcell fusion [1-3]. This technique can move the large intact genomic structures of natural chromosomes or artificially engineered chromosomes, and transferred chromosomes can be stably retained and freely segregate in recipient cells. Taking advantage of these features, MMCT has been employed very successfully in various basic science studies, e.g., genetic mapping and identification of tumor suppressor genes, analysis of genomic imprinting and production of animal models of disease [4-7]. Furthermore, MMCT is also used in gene transfer using a human artificial chromosome (HAC), mini-chromosome vector. HACs have several unique characteristics as gene-delivery vectors, including stable episomal maintenance in mammalian cells, the capacity to carry large transgenes, and less susceptibility to gene silencing, and have been applied to gene therapy [8-10], gene function analysis [11], animal transgenesis [12,13] and protein production [14-16].

In microcell fusion, protocols combining treatment of cells with phytohemagglutinin-P (PHA-P) to adhere microcells to recipient cells and fusion using polyethylene glycol (PEG) are most common, because they have proved to be more simple and efficient than those initially using inactivated Sendai virus [17]. Nonetheless, a yield frequency of microcell hybrids by PEG-induced fusion is no more than 1 × 10^-6^ – 1 × 10^-5^ [18]. Higher concentrations of PEG can produce larger numbers of fused cells, but in the meantime PEG cytotoxicity increased. Little is known about the functional mechanism of PEG, but PEG may incur a redistribution of intramembrane molecules within the plasma membrane in a cell-type dependent fashion. Therefore, it may be difficult to separate fusogenic function from cytotoxicity of PEG. To overcome the drawback of PEG-induced microcell fusion, we have developed a novel method for MMCT where Measles Virus (MV) envelope proteins (MV-MMCT) are applied instead of using PHA-P and PEG [19]. It was demonstrated that higher efficiency of microcell fusion was achieved in some human cells by means of microcells which expressed MV-derived fusion machinery, both the hemagglutinin (H) protein and fusion (F) protein, as compared to PEG-induced fusion. However, the human fibroblast cell line HFL-1 did not exhibit susceptibility to MV-derived fusion machinery. Since cellular tropism of MV is determined by binding of the H protein to cell surface protein, CD46 or SLAM [20-22], in order to extend the cell spectrum eligible for MV-MMCT, further modification of MV-derived fusion machinery is needed. It has been demonstrated that the tropism of MV can be retargeted to many different cell surface molecules by utilizing a fusion protein consisting of single-chain antibodies (scFv), peptides, growth factors or cytokines fused to the extracellular C terminus of the H protein [23]. Furthermore, co-transfection of plasmids, encoding the chimeric H protein fused to scFv which recognizes muscle-specific integrin α7 and the F protein, into human fibroblasts could induce fusion to differentiated mouse myotubes without viral infection [24]. Therefore, considering that the complex of the chimeric H protein and the F protein can induce whole cell fusion, we were keen to study whether the chimeric H protein was also capable of mediating microcell fusion.

Here we report a targeted MV-MMCT approach to transfer a HAC vector from CHO donor cells into normal human fibroblasts. The chimeric H protein was produced by fusing anti-transferrin receptor (TfR) scFv to the C-terminus of the H protein. Successfully, co-transfection of plasmids encoding the chimeric H protein and the F protein into CHO cells, which harbor the HAC vector containing drug-resistant genes, GFP and the reprogramming gene cassette for human cells, followed by MMCT, induced microcell fusion to human HFL-1 cells. Drug-resistant and GFP positive colonies were obtained after drug selection, and introduction of the HAC vector to HFL-1 cells was confirmed by FISH analysis. Transgenes from the reprogramming cassette were efficiently transcribed, and subsequently dedifferentiation occurred in some of these cells as we previously demonstrated in the case of mouse cell reprogramming [25]. This result indicated that targeted MV-derived fusion machinery had no influence on HAC functions, in fact the intact HAC could be transferred into recipient cells via targeted MV-MMCT. Taken together, targeted MV-MMCT may be powerful in extending the cell spectrum for gene transfer via HAC vectors and/or chromosome transfer; so far PEG-induced microcell fusion has been effective in only a limited number of cell types.

## Methods

### Cell culture

Human fibrosarcoma (HT1080) cells were grown in Dulbecco’s modified Eagle’s medium (DMEM) (Sigma) plus 10% fetal bovine serum (FBS). HFL-1 cells (RCB0521, RIKEN, Tsukuba, Japan) were grown in Ham’s F12 medium (Wako Pure Chemical Industries) supplemented with 15% FBS. Hprt-deficient Chinese hamster ovary (CHO) cells (JCRB0218, JCRB Cell Bank, Japan) containing the HAC vector were cultured in Ham’s F-12 medium supplemented with 10% FBS and 8 μg/ml Blasticidin S (Bsd, Funakoshi). HFL-1 cells introduced the HAC vector for reprogramming (designated as iHAC) were maintained on mitomycin-C (Kyowa Hakko Kirin)-treated SNL (STO) feeder cells (SANGER Institute, Cambridge, UK) in hES cell maintenance medium, which consisted of a 1:1 DMEM and Ham’s F-12 (Sigma) supplemented with 2 mM L-glutamine, 1 mM sodium pyruvate, 1% MEM non-essential amino acids, 0.1 mM 2-mercaptoethanol (Gibco), 4 ng/ml human basic fibroblast growth factor (Wako Pure Chemical Industries) and 20% Knockout serum replacement (Gibco).

### Flow cytometry analysis

Cells were dispersed by treatment with 0.2% EDTA/PBS, washed twice with PBS, and resuspended in ice-cold PBS containing 2% (w/v) BSA at a concentration of 10^6^ cells/ml. The cells were then incubated for 60 min on ice with a 1:50 final dilution of PE-labeled anti-TfR antibody (BD Pharmingen), or PE-labeled isotype control (BD Pharmingen). After washing with BSA/PBS, the cells were analyzed with a Gallios flow cytometer (Beckman Coulter).

### MV envelope protein expression plasmids and transfection

DNA sequence encoding the scFv recognizing transferrin receptor (TfR) was generated by PCR amplification, with scFv-expressing vector clones [26] as the template, using the following primers: 5′-GCG GCC CAG CCG GCC ATG G-3′ and 5′-CTT GCG GCC GCA CCT AGG ACG GTC AGC TT-3′. The SfiI/NotI-digested PCR products were subcloned into pTNH6-Haals [27] at the corresponding restriction sites, resulting in pTNH6-HaalsαTfR-#1-8, respectively (Additional file 1 Table S1). A BsiWI-fragment of pCAG-T7F [19] was inserted into the BsiWI site of pVITRO1-neo-mcs (InvivoGen), resulting in pVF#9. A BsrGI-fragment of pTNH6-HaalsαTfR-#5 was inserted into the BsrGI site of pVF#9, resulting in pVF#9-TfR.

Plasmids were linearized by restriction digestion with PvuI (NEB) before transfection. HAC donor CHO cells (8 × 10^4^/well in 24-well plates (Nunc)) were co-transfected with 0.3 μg each of pTNH6-H and pCAG-T7-F, and 0.25 μg of pDsRed-Monomer-N1 (Clontech) using Lipofectamine 2000 (Invitrogen). At 24 h after transfection, the cells were re-plated at low density and selected for 14 days with 800 μg/ml of G418 (Nacalai). Drug-resistant cells were recovered as a mixed population. HT1080 cells (2 × 10^6^/6 cm dish) were co-transfected with 0.4 μg each of pTNH6-H and pCAG-T7-F. After culture for 6 h, syncytium formation was tested under the microscope.

### Construction of HAC vectors for reprogramming (iHAC)

A SalI fragment of a human P53-knockdown construct (pMKO.1 puro p53 shRNA2, Addgene) was cloned into the SalI site of pinsB3 [25], resulting in pinsB3hP53sh. Finally, an AscI-SpeI fragment of pinsB3hP53sh was inserted into the AscI-NheI site of pPAC-2CAG-O2 (carrying two copies of CAG-driven Klf4, c-Myc, Sox2, and four copies of CAG-driven Oct4), resulting in pPAC-2CAG-O2hP53sh.

The reprogramming cassettes were introduced into 21HAC2 vectors [28] using the Cre-loxP system. Cre-recombinase expression vectors (pBS185; Invitrogen) (1 μg) and pPAC-2CAG-O2hP53sh (8 μg) were co-transfected into CHO/21HAC2, which are Hprt-deficient CHO (hprt^-/-^) cells carrying a 21HAC2, in a 60-mm dish using Lipofectamine 2000. Recombinant clones were selected using HAT (Sigma) and 8 μg/ml Bsd two days after transfection. After 2 weeks, drug-resistant colonies with a functional HPRT allele were identified by genomic PCR and isolated. Next, quantitative RT-PCR analysis of reprogramming factors was performed. A CHO cell donor clone that stably expressed reprogramming factors comparable to CHO/iHAC2 was selected and designated CHO/iHAC/X53. FISH analysis showed that the iHAC vector was maintained independently from the host chromosomes in CHO/iHAC/X53.

### Microcell fusion

Microcell fusion was performed as described previously [19]. CHO4H6.1 M, CHO/21HAC2 and CHO/iHAC/X53 were used as microcell donor cells. Briefly, HT1080 or HFL-1 cells were fused with microcells prepared from donor cells, and on the day following PEG fusion, the cells were replated, and selected with 3 μg/ml of Bsd. In the case of MV fusion, microcells were overlaid on recipient cells and left for 24 h. After that, the cells were replated, and cultured for 14 days in the presence of 3 μg/ml of Bsd.

### FISH analysis

FISH analyses were performed using either fixed metaphase or interphase spreads of each cell hybrid using digoxigenin-labeled (Roche) alphoid DNA probe p11-4 [29] and biotin-labeled (Roche) pPAC-2CAG-O2hP53sh, essentially as described previously [30]. Chromosomal DNA was counterstained with DAPI (Sigma). Images were captured using the Axio Imager-Z2 (Carl Zeiss).

### RT-PCR analysis

Total RNA was extracted with Trizol (Invitrogen) and treated with a Turbo DNAfree kit (Ambion) to remove genomic DNA contamination. cDNA was synthesized using an oligo(dT) primer and ReverTra Ace (Toyobo). Quantitative RT-PCR was performed using the Power SYBR Green PCR Master Mix (Applied Biosystems) on an ABI7900HT (Applied Biosystems). Semi-quantitative RT-PCR was performed with cDNA using ExTaq (Takara Bio). GAPDH and NAT1 were used as internal controls. Primer sequences are listed in Additional file 1 Table S2.

## Results

### Construction and validation of Haals-αTfR

**Figure 1:**
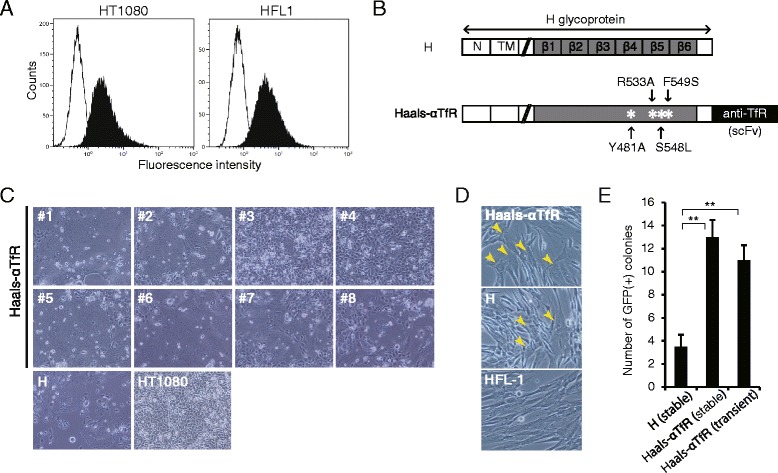
Membrane-fusion activity of Haals-αTfR in recipient cells. **(A)** Detection of surface expression of target receptors in recipient cells. Surface expression of TfR in HT1080 and HFL-1 cells was analyzed with flow cytometry by staining with PE-conjugated anti-TfR antibody (black peak) or an isotype control (white peak). **(B)** Schematic representation of recombinant H protein. scFv is displayed as a C-terminal extension of H glycoprotein. N; Amino-terminal cytoplasmic tail, TM; Transmembrane domain, *; Y481A, R533A, S548L and F549S mutations in H protein. **(C)** Syncytium formation ability differed among scFv clones. HT1080 cells were co-transfected with F and indicated H expression plasmid, and were photographed 30 hr later. **(D)** Fusion test by co-culture assay of donor and recipient cells. CHO cells stably expressing the F and H proteins were co-cultured with HFL-1, and were photographed 24 hr later. Yellow arrows indicate syncytia. **(E)** The number of resistant/GFP(+) colonies from HFL-1 cells by MV-MMCT using H or Haals-TfR. Data are the means of four independent experiments (±SD), **; p < 0.01 (unpaired t-test).

To explore applicability of MV-MMCT to human fibroblasts, TfR was selected as the target receptor in giving a new directivity for MV-H protein, because TfR is known to be ubiquitously expressed in all tissues [31]. Flow cytometric analysis demonstrated that TfR was highly expressed in both HFL-1 and HT1080 cells (Figure 1A), whereas CD46 was expressed only in HT1080 cells, and very rarely in HFL-1 cells [19]. Subsequently, recombinant retargeted H proteins were constructed by using 8 clones of anti-TfR scFvs (Additional file 1 Table S1), which recognized different epitopes of TfR, from the phage-display antibody library [32]. Anti-TfR scFvs were fused to the C terminus of a quadruple mutated H protein (Haals: Y481A, R533A, S548L and F549S), which lacked the ability to bind both CD46 and SLAM [27,33], to validate the effect of anti-TfR scFvs on cell fusion more precisely (Figure 1B). To screen 8 constructs of chimeric H proteins (Haals-αTfRs), we transfected expression plasmids encoding MV-F and Haals-αTfRs into HT1080 cells and assayed syncytium formation by homofusion. Sufficient formation of syncytia and syncytium-induced cell death was detected in clone No.1, No.5 and No.6, whereas a very low number of syncytia were formed in other clones (Figure 1C). Thus, clone No.5 (Haals-αTfR-5) was selected for use in further experiments.

Next, we tested whether chimeric MV-H proteins confer human fibroblast-directed fusion ability on CHO cells. Expression vectors encoding the MV-F, Haals-αTfR-5, and Neo^r^ were transfected into a CHO cell line containing 21HAC2 vector (CHO/21HAC2), and G418-resistant cell population was pooled, which was designated as CHO/21HAC2/TfR. As in the case with MV-F/H-transfected CHO cells [19], co-transfection with MV-F and Haals-αTfR-5 was unable to induce homofusion in CHO cells (data not shown). On the other hand, co-culture of these pooled CHO cells with HFL-1 cells caused formation of syncytium at higher frequency with the combination of MV-F/ Haals-αTfR-5 than with MV-F/H (Figure 1D). These results indicate that the recombinant Haals-αTfR-5 has a fusion activity in HFL-1 cells as well as in HT1080 cells.

### Comparison of MMCT efficiency between different protocols

To evaluate the ability of the method of Haals-αTfR-5-mediated microcell fusion, we compared the efficiency of MMCT on three different protocols, namely PEG-induced, H protein (H)-mediated, or Haals-αTfR-mediated microcell fusion. Three CHO cell line donors for each protocol were used, with CHO/21HAC2, for PEG; CHO4H6.1 M [19], for H protein; CHO/21HAC2/TfR, for Haals-αTfR. Microcells were prepared from these donor cells under standard conditions by using colcemid and cytochalasin B, followed by microcell fusion with either HT1080 or HFL-1. MMCT efficiencies were determined as the number of EGFP-positive colonies which emerged under the selective culture condition in the presence of Bsd because all donor cells carried 21HAC2 containing both EGFP and Bsd resistant genes. Haals-αTfR-mediated fusion protocol is efficacious in MMCT to HFL-1, whereas H protocol obtained the lowest number of Bsd-resistant, EGFP-positive colonies, as low as with PEG in one experiment (Table 1). In the case of HT1080, susceptible to H protocol, the Haals-αTfR protocol exhibited comparable or even greater effectiveness. Furthermore, transient expression of MV-F/Haals-αTfR provided about the same number of EGFP-positive colonies as stable expression from CHO/21HAC2/TfR (Figure 1E). These results demonstrate that retargeting of microcell fusion using Haals-αTfR could improve the efficiency of MMCT to human fibroblasts, as compared with PEG or original MV fusogen, and more convenient preparation of retargeted microcells could be achieved by transient transfection without establishment of a stable clone expressing the retargeted MV-H.

**Table 1:** **Comparison of MMCT efficiency between different protocols**

**Recipient cell**	**exp.**	**Number of GFP(+) colonies**		
		**PEG**	**H**	**Haals-αTfR**
HFL-1	1	2	3	13
	2	N/T	6	12
	3	N/T	1	10
	4	N/T	4	17
HT1080	1	15	95	215
	2	N/T	86	152

N/T; not tested.

### Capability of Haals-αTfR-mediated microcell fusion to introduce a HAC vector to human fibroblasts

**Figure 2:**
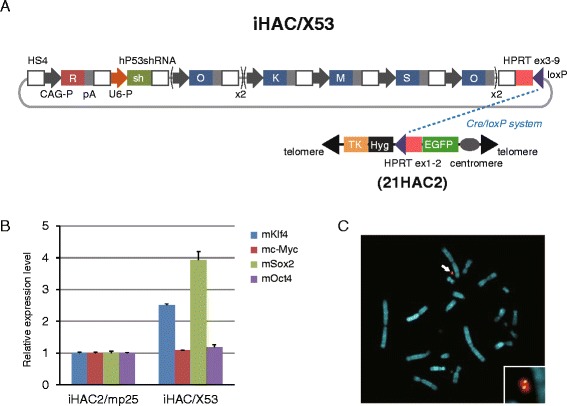
Construction of the iHAC for reprogramming human somatic cells. **(A)** Schematic diagram of the iHAC/X53. iHAC/X53 is carrying the expression cassettes for multi copies of a set of four reprogramming factors (2 copies of Klf4, c-Myc and Sox2, and also 4 copies of Oct4), a human P53shRNA construct and DsRed (R). **(B)** Expression of the four reprogramming factors contained in the iHAC vectors was confirmed by qRT-PCR. Transcript levels of transgenes were standardized to Gapdh. Transcript levels in CHO/iHAC/X53 were compared to levels in CHO/iHAC2/mp25. Error bars, s. d. **(C)** FISH analysis of CHO/iHAC/X53. Digoxigenin-labeled alphoid satellite marker (red) was used to detect the HAC backbone. Biotin-labeled pPAC-2CAG-O2hP53sh (green) was used to detect the reprogramming cassette in the iHAC. Chromosomal DNA was counterstained with DAPI. White arrows indicate the iHAC vector and the insets show enlarged images of the iHAC.

**Figure 3:**
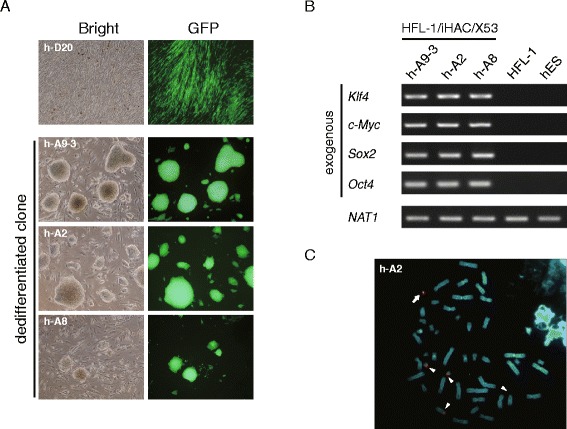
Introduction of the iHAC to HFL-1 by MV-MMCT using Haals-αTfR. **(A)** Representative bright-field and fluorescence images of an EGFP(+) colony. Three dedifferentiated clones (h-A9-3, h-A2 and h-A8) exhibited doom-like morphology, whereas a clone h-D20 still retained fibroblast-like morphology. **(B)** Expression of exogenous reprogramming factors detected by RT-PCR in EGFP(+)/dedifferentiated clones. NAT1 was used as an internal control. **(C)** FISH analysis of EGFP(+) clone. Digoxigenin-labeled alphoid satellite marker (red) was used to detect the HAC backbone and endogenous chromosomes 13 and 21. Biotin-labeled pPAC-2CAG-O2hP53sh (green) was used to detect the reprogramming cassette in the iHAC. Chromosomal DNA was counterstained with DAPI. White arrows indicate iHAC vector and the insets show enlarged images of the iHACs. Arrowheads indicate Chr.13 or 21.

To determine whether Haals-αTfR-mediated microcell fusion could be applied to transfer more functional, elaborate HAC vectors to human normal cells, we next tried human cell reprogramming by the introduction of a HAC vector via Haals-αTfR-mediated microcell fusion. The HAC vector was constructed by minor modification of iHAC2, which enables reprogramming of mouse embryonic fibroblasts to pluripotency [25], and to change mouse p53-knockdown construct into a human version (Figure 2A). The CHO cell donor clone exhibited stable and comparable expression of four factors as with CHO/iHAC2/mp25 cells and was designated CHO/iHAC/X53 (Figure 2B). FISH analysis showed that the HAC vector was maintained independently from the host chromosomes in CHO/iHAC/X53 (Figure 2C). Then, MMCT was performed from CHO/iHAC/X53 donor cells to HFL-1 recipient cells after transient expression of MV-F/Haals-αTfR in these donor cells. As seen in 21HAC2, iHAC was more efficiently transferred into HFL-1 by using the retargeted MV fusogen than PEG fusion from the objective of EGFP fluorescence (Table 2). A total 59 clones were obtained from 5 experiments about a month after MMCTs and 43 out of 59 clones were expandable up to 47 days after MMCT. Remarkably, 19% of expandable clones (9 of 43) could continue proliferating further over 2 months with morphological change to doom-like colonies resulting from tight cell-to-cell association, whereas residual clones with fibroblast-like feature gradually ceased cell proliferation (Figure 3A and Additional file 1 Figure S1). This result indicated that iHAC could dedifferentiate human fibroblasts. To assess the function of iHAC in dedifferentiation, three representative clones, h-A2, h- A8 and h-A9 subclone 3 (h-A9-3), were further analyzed. FISH analysis and semi-quantitative RT-PCR analysis indicated that all of these clones retained iHAC stably and independently from host chromosomes and consistently expressed four exogenous transgenes, constructed in the iHAC, as well as an EGFP gene (Figure 3B and C). Furthermore, the expression of endogenous pluripotent markers (SOX2 and TERT) was significantly induced in all clones, in addition to slight induction of endogenous OCT4 (Additional file 1 Figure S2). These results suggested that MMCT mediated by retargeted MV fusogen could introduce HACs into human normal cells in excellent condition without recognizable aberrations and that all of the transgenes harbored in the HAC were efficiently transcribed enough to fulfill their functions.

**Table 2:** **Summary of MMCT experiments to introduce iHAC into HFL-1**

**recipient**	**MMCT**	**exp.**	**# of GFP(+) colonies**				**Dedifferentiated clone name**
			D8-11*	D20-35*	D47-50*		
HFL-1	PEG	1	9	1	0		
	Haals-αTFR	1	N/D	26	24	(3)	hA-1, 8, 9
		2	15	5	3	(1)	hB-22
		3	25	3	2	(2)	hC-10, 29
		4	29	11	7	(1)	hD-8
		5	26	14	7	(2)	hE-10, 25

N/D; not determined, *; days after MMCT, values in parentheses denote the number of dedifferentiated clones.

## Discussion

We demonstrate that the alteration of the tropism of MV-H protein by fusing anti-TfR scFv at its C terminus is applicable to microcell fusion. This retargeted MV-H successfully induced fusion between microcells and human fibroblast HFL-1 (Figure 3 and Additional file 1 Figure S1), which was moderately hard to be recognized by original MV-H protein. These results indicate that retargeting strategy of the H protein is available for microcell fusion as well as original H protein, leading to expansion of the spectrum of target cells to which a HAC vector can be transferred. Since human normal cells, e.g. fibroblasts and mesenchymal stem cells, show less efficiency of microcell fusion by PEG than malignant cells, microcell fusion mediated by MV-derived fusion machinery is an attractive technique for gene delivery which involves the transfer of a HAC vector from a donor cell to human normal cells [14,19,34]. However, the number of GFP-positive colonies yielded from HFL-1 by retargeted MV-MMCT was approximately one tenth of that from HT1080, even though TfR was expressed on cell surface at an equivalent level in both cell lines (Figure 1A and Table 1). This difference in efficiency may be due to predisposition toward cellular quiescence of HFL-1 rather than HT1080, because many GFP-positive HFL-1 cells were detected on the day following retargeted MV-MMCT (data not shown). Indeed, in the case of iHAC introduction into HFL-1, the number of GFP-positive colonies gradually decreased from the early stage (D8-11) to the later stage (D47-50) (Table 2).

Additionally, it remains to be elucidated whether MV-mediated microcell fusion affects physiological functionality of transgenes carried on HACs; except for a drug-resistant gene and an EGFP gene, transgenes have not been assessed for their expressions or gene functions after MV-mediated microcell fusion. Here we demonstrated that transgenes of Klf-4, c-Myc, Sox2, and Oct4, were persistently expressed in EGFP-positive, iHAC-retained fibroblast clones over three months, and some of these clones exhibited dedifferentiated phenotypes: potent growth ability, marked change of cell morphology, and activation of endogenous pluripotency-related genes (Figure 3 and Additional file 1 Figure S2). Furthermore, pluripotent cells were derived from one of the dedifferentiated clones during further prolonged culture, resulting from elimination of transgene expression by spontaneous loss of iHAC (Additional file 1 Figure S3). These results were consistent with our previous study of reprogramming of mouse embryonic fibroblasts which received iHAC2 by PEG-induced microcell fusion [25]. Taken together, it can be said that MV-derived fusion machineries exhibit no influence on not only expression of transgenes carried on HACs, but on overall functions of HACs themselves, identically thus to PEG-induced fusion.

## Conclusions

In this study, we demonstrated that MV-H protein, retargeted by adding anti-TfR scFvs, yielded the expansion of cell range applicable for gene transfer via HAC vectors without perturbation of HAC functions. So far, we have demonstrated the following advantages of HAC vector systems for gene transfer over others: no size limitation of inserted genes, persistent and stable expression of these genes, and no scar in the host genome. Therefore, we anticipate that this technology applying MV fusogen will facilitate the systematic cell engineering by HACs for modification of a variety of cell types, such as reprogramming cells.

## Additional file

Additional file 1: Figure S1. Bright-field and fluorescence images of dedifferentiated EGFP(+) clones. **Figure S2.** Expression of pluripotency markers detected by RT-PCR in dedifferentiated clones. **Figure S3.** Establishment of iHAC-free human iPS cells. **Table S1.** Anti-TfR mAbs for cloning scFv-expressing vectors. **Table S2.** Primers used for PCR analysis.

## Abbreviations

HAC: Human artificial chromosome
iHAC: HAC for reprogramming
MMCT: Microcell-mediated chromosome transfer
MV: Measles Virus
PEG: Polyethylene glycol
TfR: Transferrin receptor
scFvs: single chain antibody
PHA-P: Phytohemagglutinin-P
Bsd: Blasticidin S
FISH: Fluorescence in situ hybridization
