# Supplementary material for: Retargeting of microcell fusion towards recipient cell-oriented transfer of human artificial chromosome
Source: BMC Biotechnol. 2015 Jun 19;15:58. doi: 10.1186/s12896-015-0142-z (PMC4472177; doi:10.1186/s12896-015-0142-z)
Supplement: Additional file 1: Figure S1. — Bright-field and fluorescence images of dedifferentiated EGFP(+) clones. Figure S2. Expression of pluripotency markers detected by RT-PCR in dedifferentiated clones. Figure S3. Establishment of iHAC-free human iPS cells. Table S1. Anti-TfR mAbs for cloning scFv-expressing vectors. Table S2. Primers used for PCR analysis. [file 12896_2015_142_MOESM1_ESM.pdf]

Additional Files

Supplementary Methods

M-FISH analysis

Procedures for the denaturation of metaphase chromosomes and 24XCyte mFISH probe kit (MetaSystems), hybridization, post-hybridization washes and fluorescent staining were performed in accordance with the manufacturer’s instructions. Metaphase images were captured digitally with a cooled CCD camera equipped with an ISIS mFISH software program (MetaSystems), processed and stored for subsequent analysis.

Teratoma formation

To produce teratomas, 1 x 10^6 cells were inoculated into testes of severe combined immunodeficiency mice (Charles River). After 9-13 weeks, resected teratomas were fixed in 20% formalin and processed for paraffin sectioning, then stained with hematoxylin and eosin.

Supplementary Figure 1

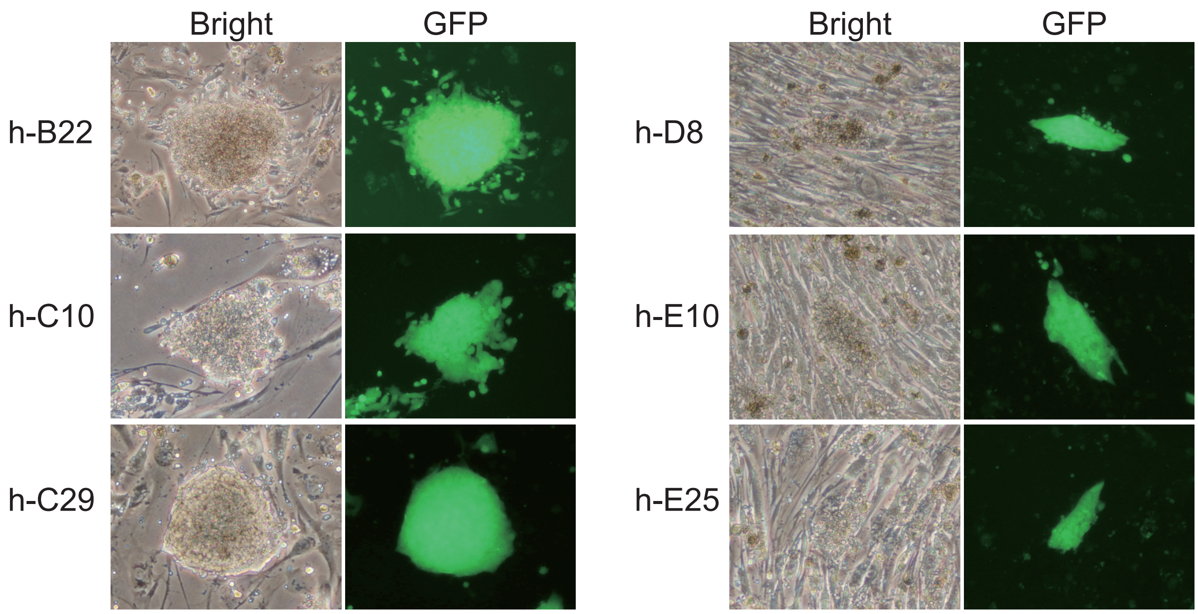

Supplementary Figure 1. Bright-field and fluorescence images of dedifferentiated EGFP(+) clones.

All clones exhibited doom-like, but not human ES-like, morphology.

Supplementary Figure 2

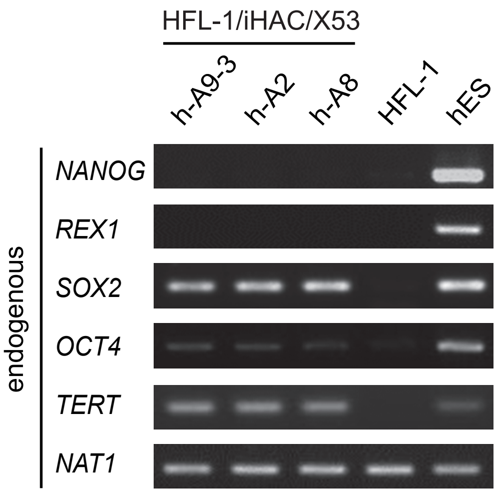

Supplementary Figure 2. Expression of pluripotency markers detected by RT-PCR in dedifferentiated clones.

Representative samples from human ES cells and unmanipulated fibroblasts (HFL-1) are also shown for comparison. NAT1 was used as an internal control.

Supplementary Figure 3

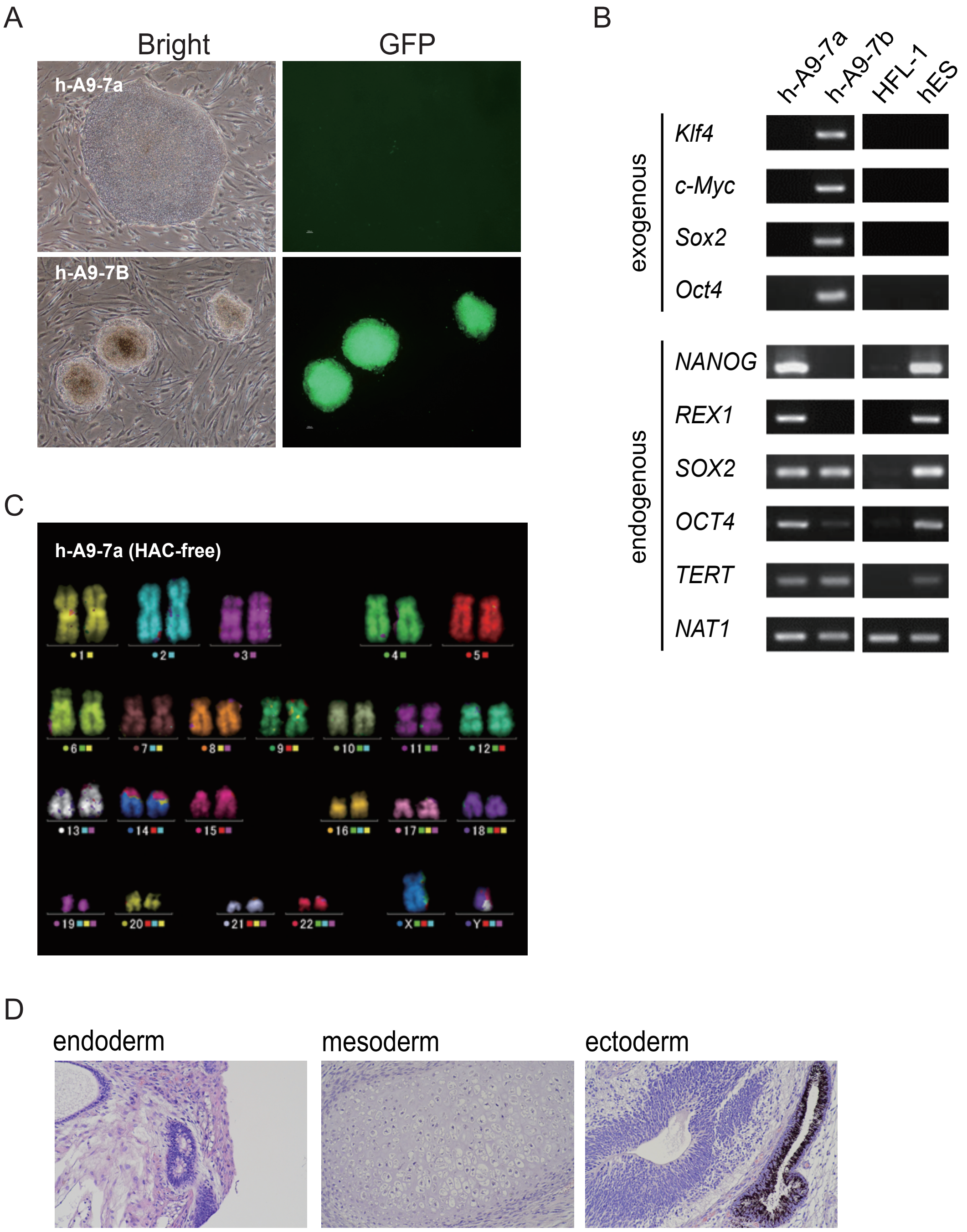

Supplementary Figure 3. Establishment of iHAC-free human iPS cells.
(A) Representative bright-field and fluorescence images of two subclones derived from h-A9. The GFP(-) clone (h-A9-7a) exhibited human ES-like morphology, whereas the GFP(+) clone (h-A9-7b) preserved doom-like morphology. (B) RT-PCR of exogenous reprogramming factors and pluripotency markers. All of endogenous pluripotent markers examined were activated in h-A9-7a up to the same level as in human ES cells. Meanwhile, none of the exogenous reprogramming factors were detected in this clone. NAT1 was used as an internal control. (C) Cytogenetic analysis of clone h-A9-7a. M-FISH analysis indicated that clone h-A9-7a had no more iHAC, and thereby had a normal karyotype. (D) Representative images of various tissues present in teratomas derived from clone h-A9-7a. Teratomas consisted of all three embryonic germ layers, endoderm, mesoderm and ectoderm, indicating that clone h-A9-7a is pluripotent.

Supplementary Table 1. Anti-TfR mAbs for cloning scFv-expressing vectors.

Vector No	anti-TfR mAbs clones
1	028-179
2	031-019
3	041-288
4	052-134
5	052-138
6	066-188
7	092-016
8	092-181

Supplementary Table 2. Primers used for PCR analysis.

Use	Primer	Sequence
HPRT genomic PCR	HPRT400F	TGGAGGCCATAAACAAGAAGA
	HPRT400R	CCTTGACCCAGAAATTCCAC
GAPDH (CHO) qRT-PCR	CHO_GAP_qRT_F	AGAAGGTGGTGAAGCAGGCAT
	CHO_GAP_qRT_R	AGGTCCACCACTCTGTTGCTGT
mKlf4 (transgene) RT-PCR	mKlf_fwd1256	GAGAAACCTTACCACTGTGAC
	CAGpA_rev1764	CAGCCACCACCTTCTGATAGG
mKlf4 (transgene) qRT-PCR	mKlf4_qRT_F1354	CTTTCAGTGCCAGAAGTGTGAC
	CAGpA_qRT_R1	ACCACCTTCTGATAGGCAGC
mc-Myc (transgene) RT and qRT-PCR	mcMyc_fwd1133	CTGCGTGACCAGATCCCTG
	CAGpA_rev1764	CAGCCACCACCTTCTGATAGG
mSox (transgene) RT and qRT-PCR	mSox_fwd778	TGGTTACCTCTTCCTCCCAC
	CAGpA_rev1764	CAGCCACCACCTTCTGATAGG
mOct (transgene) RT and qRT-PCR	mOct_fwd996	CACTCTACTCAGTCCCTTTTCCTG
	CAGpA_rev1764	CAGCCACCACCTTCTGATAGG
P53shRNA genomic PCR	HS4_F30	GCACCGCTCTTTGGAGAAGGTAAATCTTGC
	U6_Rev2548	GAAGGAATCATGGGAAATAGGCCCTC
NAT1 RT-PCR	Nat1-U283	ATTCTTCGTTGTCAAGCCGCCAAAGTGGAG
	Nat1-L476	AGTTGTTTGCTGCGGAGTTGTCATCTCGTC
NANOG RT-PCR	ECAT4-macaca-968S	CAGCCCCGATTCTTCCACCAGTCCC
	ECAT4-macaca-1334AS	CGGAAGATTCCCAGTCGGGTTCACC
REX-1 RT-PCR	hREX1-RT-U	CAGATCCTAAACAGCTCGCAGAAT
	hREX1-RT-L	GCGTACGCAAATTAAAGTCCAGA
SOX2 (endo) RT-PCR	hSOX2-S1430	GGGAAATGGGAGGGGTGCAAAAGAGG
	hSOX2-AS1555	TTGCGTGAGTGTGGATGGGATTGGTG
OCT4 (endo) RT-PCR	hOCT_end_fwd	CTCACTTCACTGCACTGTACTCCT
	hOCT_end_rev	CATTGAACTTCACCTTCCCTCC
TERT RT-PCR	hTERT_RT_F	CGGAAGAGTGTCTGGAGCAA
	hTERT_RT_R	GGATGAAGCGGAGTCTGGA
